# A case report of fungal endocarditis presenting with low back pain as the initial symptom

**DOI:** 10.1097/MD.0000000000040962

**Published:** 2024-12-13

**Authors:** Haixia Xu, Hang Zhang, Jiacheng Wu, Libo Jin

**Affiliations:** a Department of Cardiovascular Surgery, Shaoxing People’s Hospital, Shaoxing, Zhejiang Province, China.

**Keywords:** case report, diagnosis, fungal endocarditis, therapy

## Abstract

**Rationale:**

Fungal endocarditis (FE) is a rare form of infective endocarditis. Compared to bacterial endocarditis, FE develops more slowly and insidiously, with nonspecific clinical manifestations, making diagnosis more challenging. Cases presenting with low back pain as the initial symptom are exceedingly rare, leading to a high risk of misdiagnosis or delayed diagnosis.

**Patient concerns:**

A 61-year-old male was admitted due to recurrent low back pain accompanied by fever for 2 months. He had no history of invasive procedures or immunosuppressive therapy.

**Diagnoses:**

The patient was diagnosed with FE.

**Interventions:**

The patient underwent surgical treatment, during which the excrescence was removed, and mitral valve replacement was performed. Postoperatively, he received a full course of antifungal therapy.

**Outcomes:**

Postoperatively, the patient experienced relief from low back pain and was afebrile. He was discharged after completing antifungal treatment and, upon follow-up after 1 year, had no recurrence of low back pain.

**Lessons:**

In this case, the patient initially presented with low back pain, which, despite the presence of fever and other signs of infection, did not readily suggest a cardiac etiology. This case highlights the importance of not being misled by superficial symptoms and underscores the need for comprehensive and accurate physical examinations and targeted investigations for proper diagnosis.

## 1. Introduction

Fungal endocarditis (FE) is a rare but serious form of infective endocarditis, accounting for approximately 1% to 3% of all cases.^[1]^ Its rarity, coupled with a typically insidious onset and high mortality rate, poses a substantial challenge in clinical practice. Patients with FE often have underlying risk factors, such as intravenous drug use, immunosuppression, invasive procedures, or prolonged antibiotic therapy.^[2,3]^ FE typically has an insidious onset and presents with nonspecific symptoms such as fever, fatigue, and weight loss, making it difficult to distinguish from other infectious diseases.^[4]^ Therefore, by the time it is diagnosed, the disease has reached an advanced stage, frequently presenting with complications such as damage to heart structures and systemic embolism affecting multiple organs.

This report presents a case of FE with lower back pain as the initial symptom, ultimately diagnosed as Candida parapsilosis infection. We aim to share insights into the diagnosis and treatment of FE and to provide guidance for clinicians to enhance their understanding of this condition.

## 2. Case presentation

The patient is a 61-year-old male who was admitted due to “recurrent low back pain accompanied by fever for 5 months.” Five months prior, the patient developed low back pain without any apparent cause, which was most pronounced when rising from a seated position and alleviated when standing upright. This was accompanied by low-grade fever. Lumbar spine magnetic resonance imaging (MRI) revealed diffuse abnormal signals and degenerative changes in the lumbar vertebrae. The patient had undergone multiple sessions of manual therapy with no significant improvement. One day prior to admission, an echocardiogram at an outside hospital showed enhanced echogenicity of the anterior mitral valve leaflet with vegetation formation, leading to a suspicion of infective endocarditis. For further evaluation and management, the patient presented to our hospital. His past medical history is unremarkable, with no history of invasive procedures, immunosuppressive therapy, or intravenous drug use.

**Figure 1.**
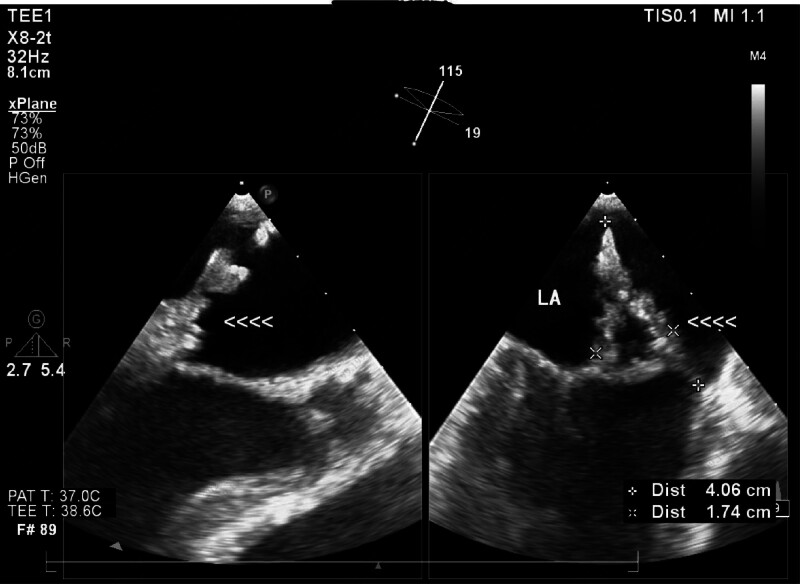
Mitral valve vegetations visible on transesophageal echocardiography.

**Figure 2.**
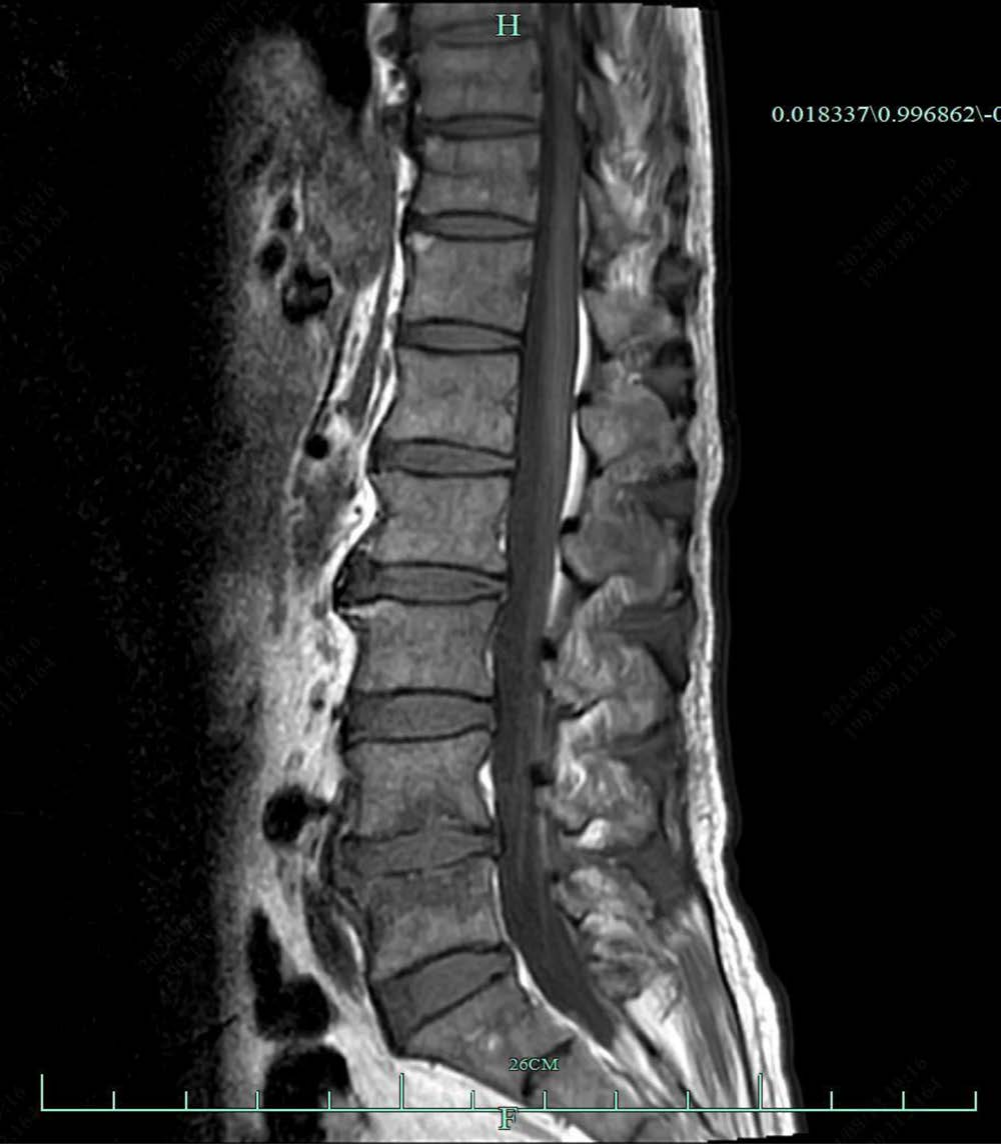
Lumbar MRI shows endplate inflammation at the lumbar vertebrae and exudative changes around the spinous processes and muscle spaces in the lower back. MRI = magnetic resonance imaging.

Physical examination: Coarse breath sounds were noted in both lungs, with no obvious dry or wet rales. The heart rhythm was regular, and a systolic blowing murmur was audible in the mitral valve auscultation area. Tenderness was present at the L4/5 intervertebral space, and there was no edema in the lower extremities.
Laboratory and auxiliary examinations: routine blood tests, coagulation profile, stool and urine analyses, biochemistry, and tumor markers were all within normal limits. Blood cultures were negative. Transesophageal echocardiography confirmed infective endocarditis, revealing mitral valve vegetation with posterior leaflet perforation and moderate to severe mitral regurgitation (Fig. 1). Lumbar MRI indicated inflammation of the L4/5 endplates, along with exudative changes around the spinous processes and muscle spaces in the lower back, suggesting an infectious process as the primary consideration (Fig. 2).
Clinical diagnosis: infective endocarditis. Following the exclusion of contraindications, the patient underwent mitral valve replacement with a mechanical valve and removal of vegetations on October 14, 2021.

The postoperative specimen culture indicated a Candida parapsilosis infection. After undergoing a complete course of antifungal treatment, the patient showed improvement and was discharged. A 1-year follow-up revealed no recurrence of symptoms like fever or low back pain. The follow-up echocardiogram revealed that the mechanical mitral valve is in the correct position and functioning normally, with no evidence of vegetations.

## 3. Discussion

FE is a rare but highly destructive form of infective endocarditis, representing approximately 1% to 3% of all cases. Unlike bacterial endocarditis, FE often has a more insidious onset and presents with less specific clinical manifestations, complicating diagnosis and treatment.

Common pathogens of FE include Candida species (such as Candida albicans), Aspergillus species, and Candida parapsilosis.^[1]^ Candida albicans is frequently associated with invasive medical procedures or device-related infections and is more commonly seen in immunocompromised patients. In this case, the patient’s medical history revealed no significant history of immunosuppression or invasive procedures, which renders the etiology more complex and atypical.

The clinical manifestations of FE are diverse and may include fever, fatigue, weight loss, heart murmurs, and peripheral signs such as skin lesions and Janeway lesions. However, these symptoms are often nonspecific. In some cases, patients may also present with symptoms like heart failure, difficulty breathing, and embolic events.^[5]^

In this case, lower back pain was the initial symptom, closely associated with spinal complications of infective endocarditis. Spinal infections, including spinal osteomyelitis, intervertebral disc inflammation, and endplate inflammation, are rare but serious complications of infective endocarditis. These conditions are typically caused by emboli detaching from the infected cardiac lesion, traveling through the bloodstream, and lodging in the spinal arteries. Although lower back pain is not a common symptom of infective endocarditis, its identification can highlight spinal infection as a crucial element in the pathophysiological process.

Rapid diagnosis and early treatment are essential to prevent severe sequelae such as heart failure, systemic embolism, and death.^[6]^ The diagnosis of infective endocarditis primarily relies on the Duke criteria, which incorporates both major and minor criteria.^[7]^ In this case, the cardiac ultrasound revealed extensive vegetation on the posterior leaflet of the mitral valve, accompanied by moderate to severe mitral regurgitation, satisfying one of the major Duke criteria. Additionally, the postoperative culture of the specimen identified a Candida infection, further confirming the diagnosis of infective endocarditis.

It is important to note that the positive rate of blood cultures in FE is relatively low, particularly if samples are obtained after the initiation of antibiotic or antifungal therapy. Therefore, when a fungal infection is suspected, it is crucial to prioritize pathological examination and culture of vegetations to enhance diagnostic accuracy. For cases where diagnosis remains elusive despite routine tests, molecular biology techniques such as PCR can be employed as supplementary diagnostic tools.^[8]^

The treatment of FE presents significant challenges and generally necessitates a combination of surgical intervention and antifungal therapy.^[1,9]^ Surgical intervention is often essential, especially for patients with complex conditions or large vegetations. Surgery not only removes infected lesions and reduces the risk of embolism but also improves cardiac function. In this case, the patient opted for mitral valve replacement surgery due to the presence of extensive mitral valve vegetation and valve perforation. Postoperative pathology confirmed that the vegetation was due to a fungal infection, underscoring the critical role of surgical resection in the management of FE.

Postoperative antifungal therapy is crucial for preventing recurrence of FE. Although *Candida parapsilosis* is generally susceptible to standard antifungal agents such as amphotericin B and fluconazole, its strong biofilm-forming ability and high resistance necessitate a prolonged treatment course and close monitoring of disease progression. In this case, the patient’s symptoms were completely resolved following surgery and antifungal treatment, with no recurrence observed during the follow-up period.

## 4. Conclusions

This case underscores the need for sustained vigilance in clinical practice when confronted with persistent fever and atypical symptoms, particularly in light of the possibility of fungal etiology. Early interdisciplinary collaboration, precise imaging studies, prompt surgical intervention, and the completion of antifungal therapy are crucial for the successful treatment of FE. This case aims to alert clinicians to thoroughly consider various potential causes in complex cases, thereby optimizing the diagnostic and therapeutic process and enhancing patient outcomes.

## Author contributions

**Conceptualization:** Haixia Xu, Jiacheng Wu.

**Investigation:** Haixia Xu, Hang Zhang, Jiacheng Wu.

**Methodology:** Haixia Xu, Hang Zhang.

**Project administration:** Hang Zhang, Libo Jin.

**Resources:** Haixia Xu, Jiacheng Wu.

**Supervision:** Libo Jin.

**Validation:** Libo Jin.

**Writing – original draft:** Haixia Xu, Hang Zhang, Libo Jin.

**Writing – review & editing:** Libo Jin.
